# Supplementary material for: Cold Ischemia Time and Graft Fibrosis Are Associated with Autoantibodies after Pediatric Liver Transplantation: A Retrospective Cohort Study of the European Reference Network TransplantChild
Source: Children (Basel). 2022 Feb 17;9(2):275. doi: 10.3390/children9020275 (PMC8870233; doi:10.3390/children9020275)
Supplement: Supplementary file 1 [file children-09-00275-s001.zip › children-1568268-supplementary.pdf]

Supplement Table S1. Center specific differences.

	Hannover n=88	Bergamo n=61	Paris n=75	Warsaw n=14	Vilnius n=11	Rome n=4	p=
LD pLTX in %	21.6%	0.0%	8.0%	85.7%	81.82%	0.0%	0.000
Split in %	77.3%	80.3%	84.0%	85.7%	100.0%	50.0%	0.260
>1 IS drug %	11.4%	26.2%	50.7%	71.4%	27.3%	75.0%	0.000
ISHAK Fibrosis Score (F0-6)	1.00/1.95/ 1.11-2.80 (n=21)	2.00/1.89/ 0.59-3.19 (n=9)	1.00/1.81/ 1.27-2.36 (n=32)	1.00/0.79/ -0.05-1.16 (n=7)	n.a.	1.75/1.75/ -1.43-4.93 (n=2)	0.500
CIT median/mean/95%CI	567/536/ 491-580 min	355/ 337/ 316-358 min	n.a.	255/295/ 231-358 min	n.a.	n.a.	0.000
Age at pLTX; median/mean/95%CI	2.00/3.02/ 2.27-3.78	1.00/2.72/ 1.71-3.74	2.00/3.60/ 2.60-4.60	1.00/2.79/ 0.16-5.41	2.00/3.27/ 0.49-6.06	9.00/8.00/ -1.64-17.64	0.359
Age at AAB analysis; median/mean/95%CI	9.00/9.00/ 7.98-10.02	5.00/6.26/ 5.06-7.46	9.00/9.14/ 6.24-12.04	6.00/7.26/ 6.08-8.43	10.00/9.55/ 5.85-13.24	13.5/12.75/ 8.57-16.93	0.001
Interval between pLTX and AAB analysis in months; median /mean/95%CI	66.00/71.52/ 61.13-81.91	32.00/39.98/ 31.89-48.08	35.00/42.88/ 35.37-50.39	59.50/74.43/ 40.10-108.75	52.00/74.91/ 34.15-115.67	57.50/57.00/ -4.23-118.23	0.000
ANA positive (>1:40)	43 (48.86%)	11 (18.03%)	5 (6.76%)	5 (38.46%)	0 (0%)	2 (50%)	
ANA > 1:80	24 (27.27%)	11 (18.03%)	4 (5.41%)	1 (7.69%)	0 (0%)	2 (50%)	
SMA positive (>1:20)	82 (93.2%)	26 (42.63%)	10 (13.51%)	10 (71.43%)	0 (0%)	3 (75%)	
SMA > 1:80	5 (5.68)	0 (0%)	1 (1.35%)	3 (21.43%)	0 (0%)	0 (0%)	

pLTX= pediatric liver transplantation, LD=living donor donation, IS=immunosuppressive, CR= chronic rejection, CIT= cold ischemia time, , AAB= auto-antibody, CI= confidence interval, n.a.=no data available.

Supplement Table S2. ISHAK Fibrosis Score

Histological result	ISHAK Fibrosis Score
No fibrosis	**0**
Fibrous expansion of some portal areas, with or without short fibrous septa	**1**
Fibrous expansion of most portal areas, with or without short fibrous septa	**2**
Fibrous expansion of most portal areas with occasional portal to portal bridging	**3**
Fibrous expansion of portal areas with marked bridging (portal to portal as well as portal to central)	**4**
Marked bridging (portal–portal and/or portal–central) with occasional nodules (incomplete cirrhosis	**5**
Cirrhosis, probable or definite	**6**

Supplement Table S3. Rejection Activity Index

Category	Criteria	Score
**Portal Inflammation**	Mostly lymphocytic inflammation involving, but not noticeably expanding, a minority of the triads	**1**
	Expansion of most or all of the triads, by a mixed infiltrate containing lymphocytes with occasional blasts, neutrophils and eosinophils	**2**
	Marked expansion of most or all of the triads by a mixed infiltrate containing numerous blasts and eosinophils with inflammatory spillover into the periportal parenchyma	**3**
**Bile Duct Inflammation Damage**	A minority of the ducts are cuffed and infiltrated by inflammatory cells and show only mild reactive changes such as increased nuclear:cytoplasmic ratio of the epithelial cells	**1**
	Most or all of the ducts infiltrated by inflammatory cells. More than an occasional duct shows degenerative changes such as nuclear pleomorphism, disordered polarity and cytoplasmic vacuolization of the epithelium	**2**
	As above for 2, with most or all of the ducts showing degenerative changes or focal lumenal disruption	**3**
**Venous Endothial Inflammation**	Subendothelial lymphocytic infiltration involving some, but not a majority of the portal and/or hepatic venules	**1**
	Subendothelial infiltration involving most or all of the portal and/or hepatic venules	**2**
	As above for 2, with moderate or severe perivenular inflammation that extends into the perivenular parenchyma and is associated with perivenular hepatocyte necrosis	**3**

The RAI Score is the sum of the 3 category scores (maximum score 9).

Apated from: Banff schema for grading liver allograft rejection: an international consensus document. Hepatology. 1997 Mar;25(3):658-63. doi: 10.1002/hep.510250328. PMID: 9049215.
